# Genome-wide analysis reveals genomic diversity and signatures of selection in Qinchuan beef cattle

**DOI:** 10.1186/s12864-024-10482-0

**Published:** 2024-06-05

**Authors:** Hengwei Yu, Ke Zhang, Gong Cheng, Chugang Mei, Hongbao Wang, Linsen Zan

**Affiliations:** 1 College of Animal Science and Technology, Northwest A&F University, Yangling, Shaanxi 712100 China; 2 College of Grassland Agriculture, Northwest A&F University, No.22 Xinong Road, Yangling, 712100 China; 3 National Beef Cattle Improvement Center, Yangling, 712100 China

**Keywords:** Qinchuan cattle, Population structure, Selection signatures, Genome-wide sequencing

## Abstract

**Background:**

Indigenous Chinese cattle have abundant genetic diversity and a long history of artificial selection, giving local breeds advantages in adaptability, forage tolerance and resistance. The detection of selective sweeps and comparative genome analysis of selected breeds and ancestral populations provide a basis for understanding differences among breeds and for the identification and utilization of candidate genes. We investigated genetic diversity, population structure, and signatures of selection using genome-wide sequencing data for a new breed of Qinchuan cattle (QNC, *n* = 21), ancestral Qinchuan cattle (QCC, *n* = 20), and Zaosheng cattle (ZSC, *n* = 19).

**Results:**

A population structure analysis showed that the ancestry components of QNC and ZSC were similar. In addition, the QNC and ZSC groups showed higher proportions of European taurine ancestry than that of QCC, and this may explain the larger body size of QNC, approaching that of European cattle under long-term domestication and selection. A neighbor-joining tree revealed that QCC individuals were closely related, whereas QNC formed a distinct group. To search for signatures of selection in the QNC genome, we evaluated nucleotide diversity (θπ), the fixation index (*F*_ST_) and Tajima’s D. Overlapping selective sweeps were enriched for one KEGG pathway, the apelin signaling pathway, and included five candidate genes (*MEF2A*, *SMAD2*, *CAMK4*, *RPS6*, and *PIK3CG*). We performed a comprehensive review of genomic variants in QNC, QCC, and ZSC using whole-genome sequencing data. QCC was rich in novel genetic diversity, while diversity in QNC and ZSC cattle was reduced due to strong artificial selection, with divergence from the original cattle.

**Conclusions:**

We identified candidate genes associated with production traits. These results support the success of selective breeding and can guide further breeding and resource conservation of Qinchuan cattle.

**Supplementary Information:**

The online version contains supplementary material available at 10.1186/s12864-024-10482-0.

## Background

Cattle are one of the most successfully domesticated animals and have had an inseparable connection with human civilization since ancient times [1]. In ancient times, cattle played a vital role in ploughing in agriculture and transportation; in modern times, they remain a valuable source of high-quality protein. Artificial selection has led to remarkable changes in the size and appearance of beef cattle, leaving imprints on their genomes [2]. The identification of these genomic alterations and their application in modern molecular breeding can accelerate progress in beef cattle breeding. Whole-genome sequencing has emerged as a powerful tool for evaluating population structure and identifying specific genetic variants affecting complex agricultural traits, such as environmental adaptation, meat quality, and disease resistance [3–5].

China alone is home to 55 local cattle breeds, among which Qinchuan, Yanbian [6], Luxi [7], Jinnan [8], and Nanyang [9] are the most well-known. These indigenous cattle breeds have played a significant role as a major labor force in agriculture for thousands of years and gradually became a high-quality ingredient in traditional Chinese cuisine [10]. Qinchuan cattle (QCC) is a major indigenous breed in China, named for the Guanzhong Plain, Shaanxi Province. It is renowned for its excellent meat quality [11], survival on cultivated land, and adaptability to poor natural conditions. However, it has a few limitations, such as an underdeveloped hind hip and a slow growth rate [2]. Extensive research has focused on QCC, leading to continuous improvements in selection and breeding. As a result of this research, a new strain, Qinchuan cattle (QNC) has been successfully bred, with improvements in body size, appearance, and production performance [12]. Genomic variation has unselected Qinchuan cattle and the QNC [2]. However, the recent application of high-density chips to Qinchuan cattle has altered the breeding process significantly. Accordingly, further comparative analyses of breeds are needed. Zaosheng cattle (ZSC) are concentrated in Qingyang City, Gansu Province, China. Historical records show that people in the Zaosheng area have been selectively breeding Qinchuan cattle from the Guanzhong area of Shaanxi Province since 490 AD. This has led to the formation of a larger local population of cattle, referred to as “Dongniu”. ZSC are similar in body shape and appearance to Qinchuan cattle, and their coats are mainly red and purple (https://www.nahs.org.cn/zt_10027/xqycpc/). Horeover, the National Breed List of Livestock and Poultry Genetic Resources (2021 edition) published by the Ministry of Agriculture and Rural Affairs, PRC reported substantial genetic differentiation between QCC and ZSC, which were classified as separate local breeds. Therefore, the classification of ZSC genetic resources has been unclear. QNC and ZSC are the products of artificial selection on the original Qinchuan cattle, with consistent traits, such as coat color, resistance to rough feeding, and adaptability. However, QNC is superior with respect to meat performance. In the process of QNC breeding, it is worth determining the particular loci under strong selection.

In this study, we performed whole-genome resequencing using 21 QNC, 20 QCC and 19 ZSC samples. Additionally, we evaluated sequencing data for representative commercial and native breeds distributed worldwide downloaded from the GEO database and pre-laboratory sequencing data for Wenling and Leiqiong cattle to identify single nucleotide polymorphisms (SNPs) and investigate population structure, genomic diversity, and traits. The results of this study will serve as a foundation for further research on the genetic underpinnings of key economic traits at the genome-wide level and offer insights to facilitate the efficient implementation of cattle breeding programs.

## Results

### Whole genome re-sequence and SNP identification

**Fig. 1:**
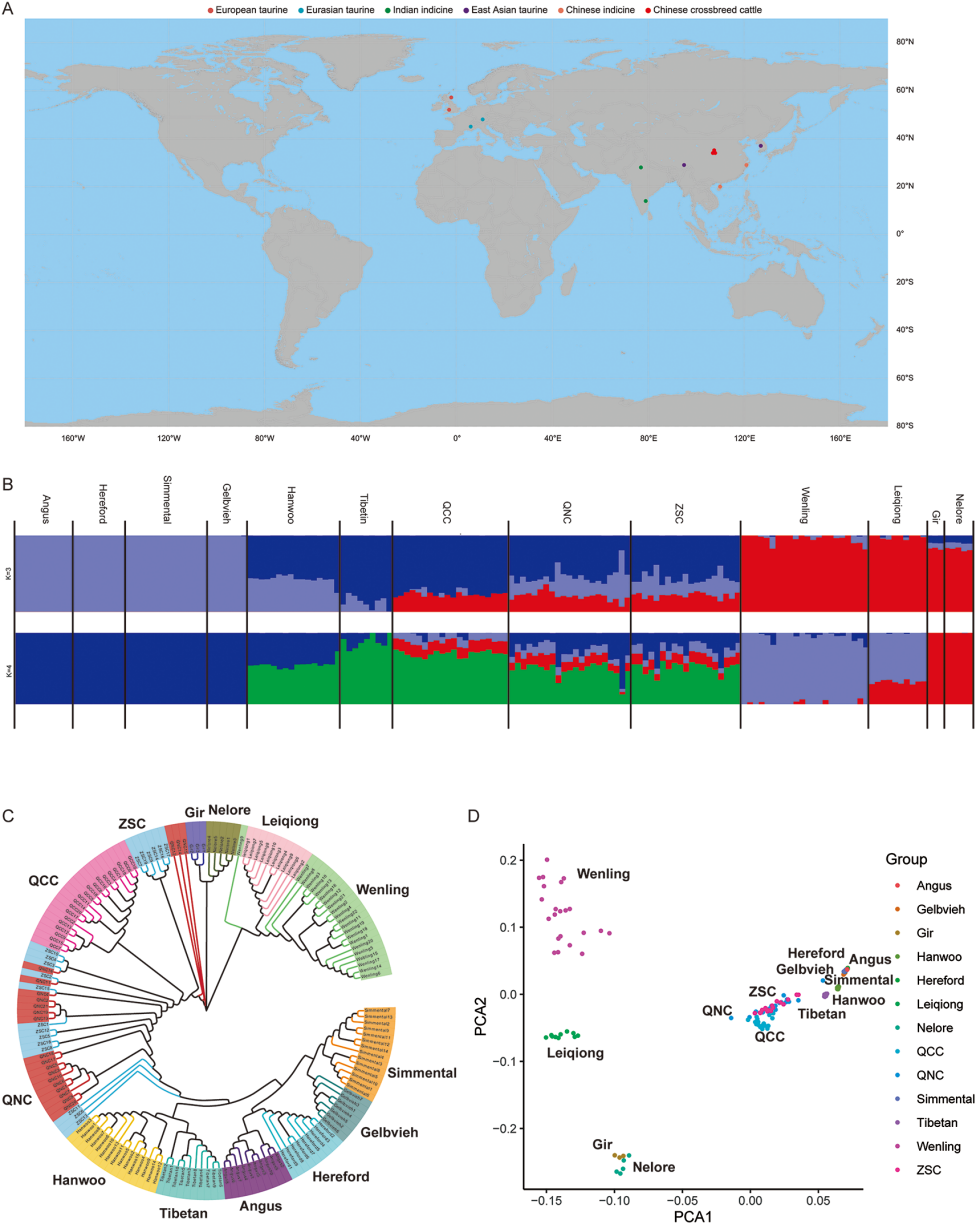
Population structure and relationships among 13 populations. (**A**): Geographic map showing the distribution of cattle populations included in this study. (**B**): ADMIXTURE analysis setting K = 3 and K = 4 for model-based clustering among various cattle groups. (**C**): Neighbor-joining tree of 13 cattle groups (161 animals). (**D**): Principal component analysis of 13 cattle populations

A total of 60 cattle classified as QNC, QCC and ZSC underwent genome sequencing. In total, 6,795,764,932 clean reads were obtained, with an average sequencing depth of approximately 12.9× per individual (Table S1). Samples from five “core” cattle, representing European taurine (Hereford and Angus), Eurasian taurine (Gelbvieh and Simmental), East Asian taurine (Hanwoo and Tibetan), Chinese indicine (Leiqiong and Wenling) and Indian indicine (Nelore and Gir), were also collected. A total of 165 animals were selected for genomic analyses (Fig. 1A and S2). The reads were aligned to the ARS-UCD1.2 genome using BWA-MEM (0.7.17). A total of 25,190,183 mutant loci were retained, and the data were subjected to filtering and quality control using plink based on minor allele frequency (MAF: 0.05) and locus completeness (geno: 0.8) filters, retaining only the second allele.

### Population structure and relationships

To explore the genetic relationships between three subgroups (QNC, QCC and ZSC) and other cattle breeds around the world, we performed ancestry estimation, constructed neighbor-joining (NJ) trees, and performed principal component analysis (PCA). In an ADMIXTURE analysis, we calculated the cross-validation errors (CV) for K = 1 to K = 13 (Table S3). When K = 3, the CV was minimal (0.24569), indicating the optimal grouping. Furthermore, QCC exhibited shared genomic ancestry with East Asian taurine, Chinese indicine, and European taurine. Interestingly, when K = 4, the proportions of European taurine ancestry in QNC and ZSC were higher than that in QCC, suggesting that the development of this new strain may have involved pedigree selection (Fig. 1B).

The “core” herds were formed independent groups, with the three subgroups positioned between *Bos taurus* and *Bos indicus*. Chinese indicine (Leiqiong and Wenling) and Indian indicine (Nelore and Gir) differed significantly from other groups. Interestingly, the three subgroups were clustered together; however, the 20 individuals in QCC were clustered tightly, with ZSC and QNC cattle scattered around QCC (Fig. 1C). The PCA showed that Bos indicus, crossed cattle and Bos taurus populations could be separated along PCA1, and Chinese indicine and Indian indicine were separated along PCA2 (Fig. 1D).

### Runs of homozygosity, genetic diversity and linkage disequilibrium

**Fig. 2:**
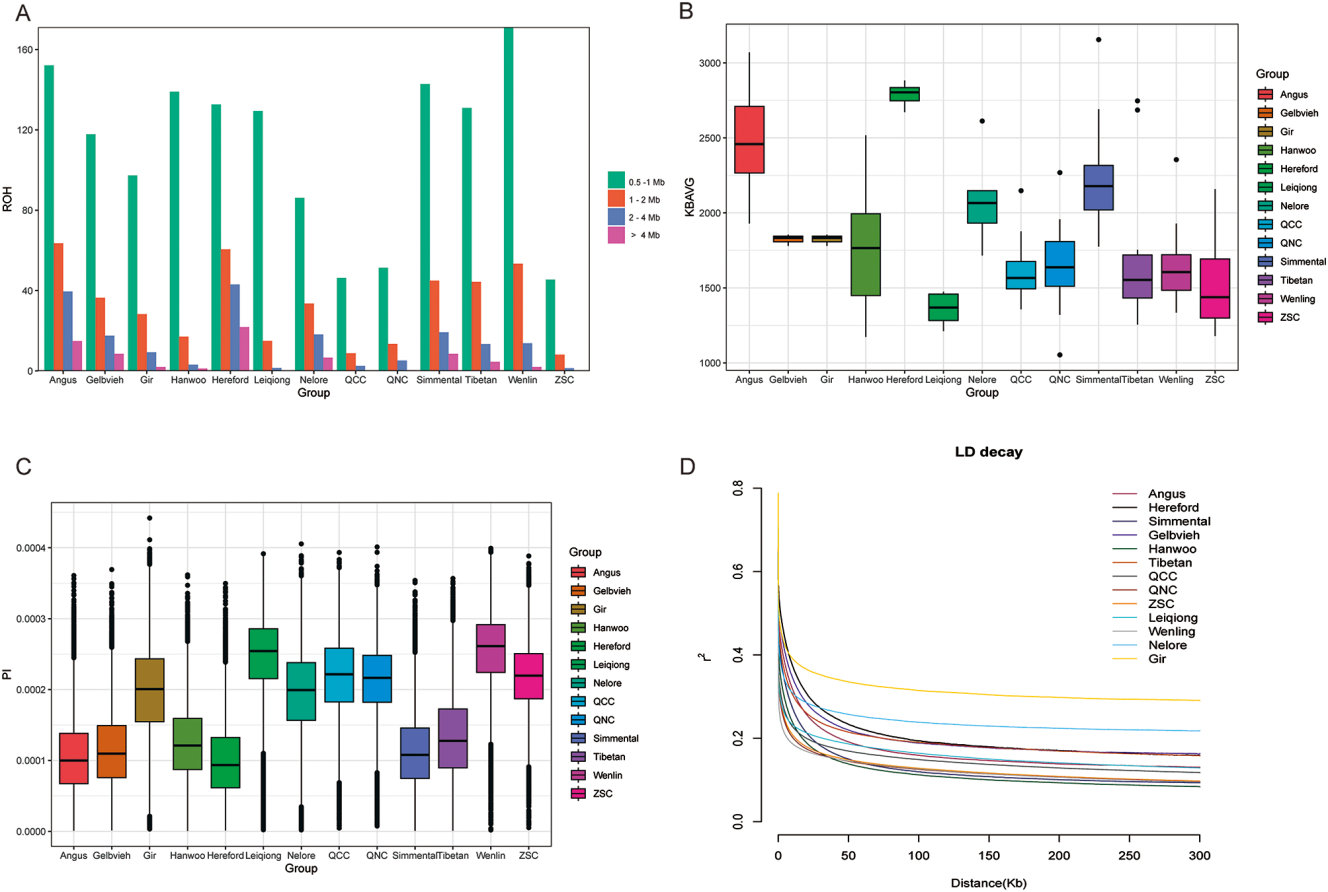
Summary statistics for genomic variation. (**A**): Distribution of runs of homozygosity (ROH) of various lengths. (**B**): Distribution of ROH in each group. (**C**): Genome-wide distribution of nucleotide diversity (θπ) in each group. The median value is indicated by the horizontal line inside the box, while the first and third quartiles are represented by the box limits. Outliers are shown as points outside the whiskers. (**D**): Genome-wide mean decay of linkage disequilibrium (LD) in each group

To assess runs of homozygosity (ROH) in three subgroups and other cattle breeds, we categorized the ROH length into four size categories: 0.5–1 Mb, 1–2 Mb, 2–4 Mb, and > 4 Mb (Fig. 2A and Table S4-5). ROH is crucial for studying the level of inbreeding and exploring population dynamics. As shown in Fig. 2A, the proportion of ROH in the 0.5–1 Mb category was highest in different groups. The proportions of longer ROH were lower; however European beef cattle breeds exhibited longer ROH segments. Among the three subgroups, QNC had a slightly higher number of longer ROH compared with those in QCC and ZSC. Figure 2B shows that the longest ROH segments were found in European taurine (Angus and Hereford), while the shortest segments were found in Chinese indicine (Leiqiong and Wenling) and local cattle (Qinchuan cattle). Additionally, the ROH segments in QNC were longer than those in QCC and ZSC, indicating a slight increase in the inbreeding level in the QNC population due to continuous breeding. In addition, we calculated the inbreeding coefficient F_ROH_. In general, of commercial cattle breeds (Hereford, 0.6309, Angus, 0.6154, Simmental, 0.5837, Gelbvieh, 0.5767, Hanwoo, 0.5517) were higher than native breeds (Tibetan, 0.4650, Nelore, 0.4191, Gir, 0.3518, Leiqiong, 0.890, and Wenling, 0.0540). F_ROH_ of QNC (0.1460) and ZSC (0.1192) were higher than that of QCC (0.0628). As shown in Fig. 2C, nucleotide diversity was lowest in specialized meat breeds, such as Hereford, Angus, and Simmental. Conversely, the nucleotide diversity was highest in Chinese indicine (Leiqiong and Wenling), followed by QNC, QCC and ZSC. QNC exhibited slightly lower genetic diversity than those of QCC and ZSC, suggesting that less polymorphism information was lost as a result of intensive selection in new Qinchuan cattle strains. Figure 2D shows that genome-wide linkage disequilibrium (LD) was low in the three subgroups, with higher LD values observed in QNC and ZSC than in QCC. In addition, Indian indicine (Nelore and Gir) exhibited the highest LD value.

### Signature of detection in the QNC and gene annotation

**Fig. 3:**
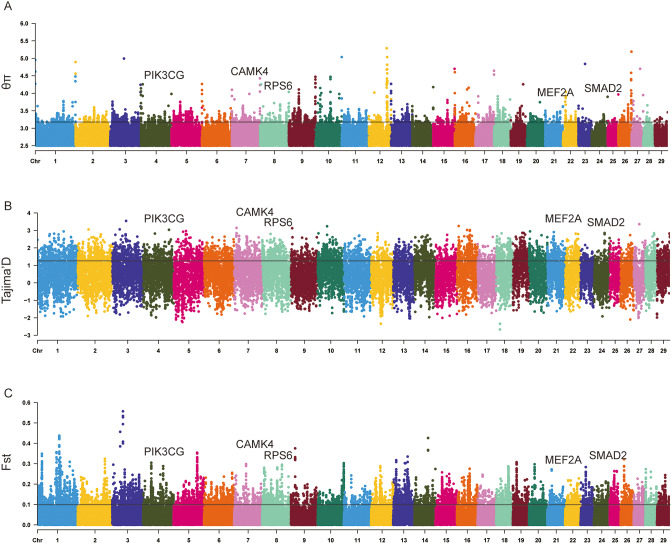
Signatures of positive selection in the genome of QNC. (**A**): Manhattan plot of θπ (setting the top 1% of values as the threshold (-log10 = 3.26)). (**B**): Manhattan plot of Tajima’s D (0.05). (**C**): Manhattan plot of *F*_ST_ between QNC and QCC (0.1)

**Fig. 4:**
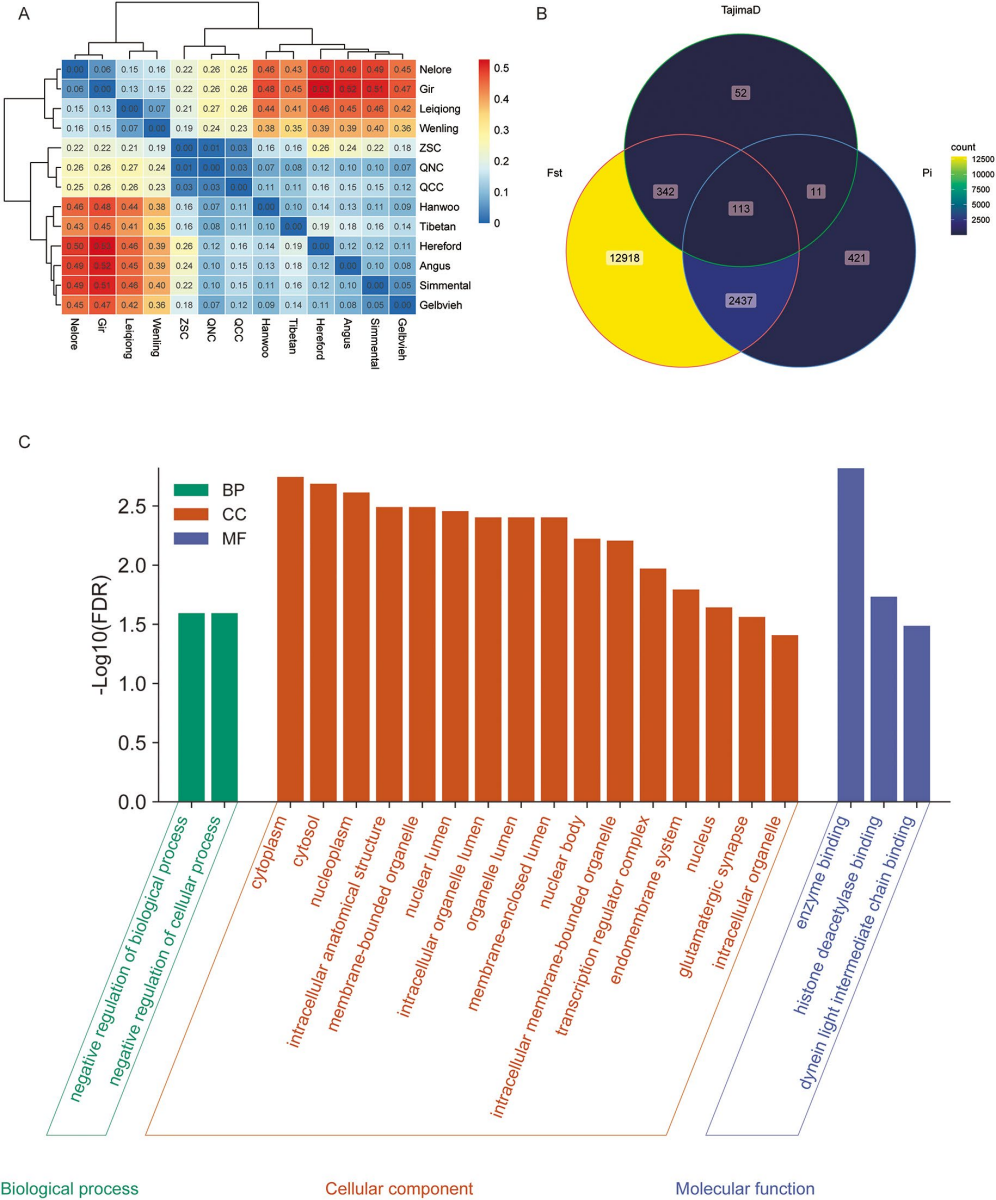
*F*_ST_ between groups and GO terms of overlapping genes in analyses of θπ, *F*_ST_ and Tajima’s D. (**A**): Heatmap of *F*_ST_ between groups. (**B**): Venn diagram showing the gene overlap based on θπ, *F*_ST_ and Tajima’s D. (**C**): GO annotation histogram of the common genes

We analyzed nucleotide diversity (θπ), fixation index (*F*_ST_), and Tajima’s D to investigate genomic signatures associated with QNC (Fig. 3 and Table S6-8). Genetic differentiation between breeds and subgroups was examined using *F*_ST_ (Fig. 4A). In general, the smallest *F*_ST_ values (0.01–0.03) were observed within subgroups (QCC, QNC and ZSC), while the largest *F*_ST_ values (0.35–0.53) were found between *Bos indicus* and *Bos taurus*. *F*_ST_ between QCC and *Bos indicus* was greater than that between European taurine and East Asian taurine and *F*_ST_ values were high between geographically distant cattle breeds. To minimize the inclusion of false-positive candidate regions, we obtained the top 1% (-log10 = 3.26) of windows for θπ and 0.05 for Tajima’s D as the selected region and defined the screening threshold for *F*_ST_ = 0.1. We then annotated the candidate ranges identified by each of the three methods individually and identified a total of 113 genes that overlapped among θπ, *F*_ST_ and Tajima’s D, indicating that these were considered regions with evidence for selective sweeps (Fig. 4B and Table S9). Using g: Profiler, 21 significant (*P*-value were corrected for multiple testing, FDR < 0.05) were enriched in QNC (Fig. 4C and Table S10). The most significant terms were “enzyme binding, GO:0019899” in the molecular function category, “negative regulation of biological process, GO:0048519” in the biological process category, and “cytoplasm, GO:0005737” in the cellular component category. We performed KOBAS for KEGG pathway enrichment [13, 14]. The shared genes were enriched in only one significant KEGG pathway, the Apelin signaling pathway (Corrected *P*-value = 0.015, Table S11). Some candidate genes associated with important traits were found (Table 1).

**Table 1:** Candidate genes associated with various phenotypes putatively under selection

KEGG pathway		ID	Candidate gene	Trait	Studies
Apelin signaling pathway		bta04371	*MEF2A*	skeletal muscle development	[15–17]
			*SMAD2*	myogenesis	[18]
			*CAMK4*	under domestication; testicular growth; oogenesis	[19, 20]
			*RPS6*	antlers grow rapidly	[21]
			*PIK3CG*		-

## Discussion

Analyses of population structure and genetic diversity provide an effective foundation for the utilization and conservation of domestic animal genetic resources. Genomic variation has been reported in Chinese local cattle breeds such as Nanyang cattle [22], Qinchuan cattle [2], and Jiaxian Red cattle [3]. The genomes of four Nanyang cattle and four Qinchuan cattle have been sequenced, revealing higher diversity in Nanyang cattle than in Qinchuan cattle [22]. Genomic differences between the original breed of Qinchuan cattle (*n* = 10) and the new strain of Qinchuan cattle (*n* = 10) have been reported in 2018 [2]. In this study, we considered group size, sex and kinship and compared genetic diversity in cattle breeds distributed worldwide to gain a more reliable overview of breed differences. The ADMIXTURE analysis revealed the ancestry of QCC were mainly from East Asian taurine, Chinese indicine and European taurine. Based on NJ trees, individuals of different breeds clustered into independent branches, and original Qinchuan cattle clustered tightly together, while individuals in the three subgroups could not be effectively separated. Artificial selection led to changes in physical characteristics and the body size index of QNC, including improvements in body height, body length, and chest circumference over those in QCC [12]. A population structure analysis revealed a close relationship between QNC and ZSC. Furthermore, both QNC and ZSC exhibited a higher proportion of European taurine ancestry than that of the original Qinchuan cattle, and this increase might contribute to the larger body size of QNC. The NJ analysis revealed a close relationship among the original Qinchuan cattle, whereas QNC showed distinct clustering patterns, consistent with our previous finding [2]. Interestingly, the PCA revealed that populations of *Bos indicus*, crossed cattle and *Bos taurus* were separated along PCA1, and Chinese indicine and Indian indicine were separated along PCA2 (Fig. 1D). This branching pattern, to some extent, reflects the geographical distribution of different cattle populations.

ROH lengths can provide insights into the history of inbreeding, where a shorter ROH reflects older inbreeding events, while a longer ROH suggests recent and close inbreeding [21]. In this study, European taurine (Angus and Hereford) exhibited the longest ROH, while Chinese indicine (Leiqiong and Wenling) and crossed cattle (QCC) showed the shortest ROH lengths. QNC had the highest frequency of long ROH (> 4 Mb), followed by ZSC, indicating a higher level of inbreeding in recent generations. This indicates that the genetic diversity in Chinese crossed cattle was higher than that in commercial varieties. Moreover, specialized meat breeds, such as Hereford and Angus, displayed the lowest nucleotide diversity levels, Chinese indicine showed the highest values, and QCC showed intermediate values. Indian indicine (Nelore and Gir) exhibited the highest LD values, while QCC showed the lowest values, in line with previous research [3]. Among the three subgroups, QNC displayed longer ROH lengths than those of QCC and ZSC, indicating a slight increase in inbreeding within QNC populations. The genetic diversity of QNC was slightly lower than that of QCC and ZSC, implying a reduction in polymorphism in QCC genomes due to artificial selection. QNC and ZSC exhibited higher LD values than those for QCC. During the breeding process in QNC, the intensive selection of bulls with favorable phenotypes resulted in an increase in population inbreeding over multiple generations. Figure 2 shows that QCC, QNC, and ZSC had similar levels of nucleotide diversity, which may be due to their similar genetic backgrounds. Additionally, the pattern of LD decay in each variety aligned with the nucleotide diversity results. Overall, the genetic diversity in Chinese local breeds was higher than that in European taurine and Eurasian taurine, consistent with previous research [3]. Compared with diversity in commercial varieties, genetic diversity in QNC was higher, suggesting that there is still enormous potential for selective breeding.

Positive selection happens when allele is preferred by the process of natural selection. Many inference methods have been developed to detect selective scanning and localize targets of directional selection in genomes. In population genetic models, demography and population structure are important influencing factors [23]. Moreover, a variety of methods and tools can be used to detect sweeps, from simple implementations that compute summary statistics such as Tajima’s D, to more advanced statistical approaches that use combinations of statistics, maximum likelihood, machine learning. Their false positive rate increases when a misspecified demographic model is used to represent the null hypothesis [24]. In addition, a new parametric test based on composite likelihood is proposed with high power to detect selective scanning and low type I error for assumptions about recombination rates and demography [25]. These new algorithm models significantly improve the accuracy of genomic scas in whole-genome sequencing data. *F*_ST_ values were low within the five core groups and slightly larger between groups. Conversely, *F*_ST_ between QCC and *Bos taurus* was approximately 0.1, while *F*_ST_ between QCC and *Bos indicus* was approximately 0.2, suggesting that the Chinese crossed breed were more closely related to *Bos taurus* and more distantly related to *Bos indicus*, consistent with the NJ analysis and PCA plots (Fig. 1B and D). Previous studies have shown that the average level of differentiation between QNC and QCC, as measured by the fixation index, was 0.017 [2]. The value obtained in this study (0.033) was around two-fold higher than this previous estimate. This indicates that the use of high-density chips in breeding significantly enhances artificial selection, supporting the crucial role of genomics in breeding. In addition, we can also use the *F*_ST_ values between populations as a reference for biological classification. *F*_ST_ values were 0.007 for QNC and ZSC and 0.031 for QCC and ZSC. These results indicate that the genomic differences between QCC and ZSC are indeed not matched across breeds. The Qinchuan cattle, the ancestors of QNC and ZSC, were artificially bred and improved, resulting in minimal differentiation between the two breeds, both of which are distinct from the original population.

We evaluated signals of selection based on nucleotide diversity (θπ), fixation index (*F*_ST_) and Tajima’s D in the Qinchuan cattle genome. The overlapping genomic regions showed enrichment for a single KEGG pathway, the apelin signaling pathway, and contained five candidate genes (*MEF2A*, *SMAD2*, *CAMK4*, *RPS6*, and *PIK3CG*). To gain insights into selective pressures, we investigated the predicted biological functions of these genes. Myocyte enhancer factor 2 A (*MEF2A*), a basic helix-loop-helix (bHLH) transcription factor, plays a crucial role in skeletal muscle differentiation [15, 16]. Our lab has examined the function of *MEF2A* in myoblast differentiation. In particular, we observed an upregulation of total MEF2A during myoblast differentiation and detected two protein bands with different molecular weights by SDS-PAGE. Furthermore, we demonstrated the involvement of the MEF2A-MEG3/DIO3-PP2A signaling cascade in myoblast differentiation [17]. Mef2a is highly expressed in satellite cells, regulates Pdha1 expression and is associated with skeletal muscle development [26]. The Smads family of proteins plays a crucial role in the TGF-β signaling pathway by transferring signals from the cell membrane to the nucleus, thereby modulating the transcription of target genes. Kinesin-mediated transport of Smad2 is required for signaling in response to TGF-beta ligands [27]. In addition, studies have shown that MicroRNA-323-3p promotes myogenesis by targeting Smad2 [18]. Notably, our lab has evaluated *Smad2* and *Smad3* at the cellular level. *C/EBPα* and *C/EBPβ* promote *Smad2* gene expression in bovine myoblasts grown in high serum growth media. In a genome-wide selection study involving wild relatives of domestic sheep, *CAMK4* was identified as a key domestication gene associated with economic traits [19]. In addition, in a genome-wide association analysis of key traits in chickens, *CAMK4* was identified as a potentially important candidate for testicular growth [20]. In a high-quality haplotype of sika deer antlers based on chromosome-scale genomes, *RPS6* was identified as one of several extended gene families that may help velvet antlers grow rapidly without causing cancer [21]. These genes may be related to the excellent meat production performance of QNC. *PIK3CG* has not been associated with economics traits in animals and poultry. We focus a lot on the enriched GO terms and KEGG pathways. The majority of these terms are very genetic and broad, which limits the interpretation of the functionality of the genes in the context of the selection sweeps observed. This may be due to the fact that we conducted the analysis between two subpopulations, and there are certain phenotypic differences between them, but the differences are not obvious enough. However, these findings provide a new perspective for studying muscle growth and development, and can explore gene functions at the cellular level.

QCC is an excellent local cattle genetic resource in China [2, 10, 22]. Extracting candidate genes associated with important economic traits is of great significance for accelerating breeding progress in QNC.

## Conclusion

In this study, genetic variation in three subpopulations related to Qinchuan cattle was evaluated using whole-genome data revealing patterns of population structure, differentiation and genomic diversity providing a basis for biological breed classification. The fixation index obtained in this study (0.033) was around two-fold higher than this previous estimate (0.017). And we identified one significant KEGG pathway, the apelin signaling pathway, as well as a series of candidate genes (*MEF2A*, *SMAD2*, *CAMK4*, *RPS6*, and *PIK3CG*) that may play a crucial role in meat-producing performance. Our findings serve as a baseline for further exploration of genomic features and intraspecific selection of local beef cattle resources.

## Materials and methods

### Ethics statement

Ethics approval for all animal experiments was granted by the Institutional Animal Care and Use Committee of Northwest A&F University (protocol number: NWAFUCAST2018-167), following the recommendations of the Regulations for the Administration of Affairs Concerning Experimental Animas of China.

### Sample collection and whole-genome sequencing

In total, 165 domestic cattle were sampled. Some samples were collected from the new strain of Qinchuan cattle (QNC, *n* = 21) of the National Beef Cattle Improvement Center’s experiment farm (Yangling, China), the original Qinchuan cattle breed (QCC, *n* = 20) of the genetic resource conservation of Qinchuan Cattle (Fufeng, China) and Zaosheng cattle (ZSC, *n* = 19) of Longshang Tianyuan Agriculture and Animal Husbandry Co., Ltd. (Zhengning, China). In addition, 105 individuals from other major beef cattle breeds were sampled of Gene Expression Omnibus database (Angus (*n* = 10), Hereford (*n* = 9), Simmental (*n* = 14), Gelbvieh (*n* = 7), Hanwoo (*n* = 15), Tibetan (*n* = 10), Leiqiong (*n* = 10), Wenling (*n* = 22), Nelore (*n* = 5) and Gir (*n* = 3)). In short, a specific breed was collected from a specific farm.

The animals were restrained in pens or neck clamp and disinfected with alcohol-cotton balls for blood collection from the jugular vein into 5mL blood collection tubes containing EDTA under a negative pressure. Samples were stored at -80 °C until use. DNA extraction, detection, fragment purification, library construction, and whole-genome sequencing to obtained 150 bp paired-end using the MGISEQ-2000 platform were performed by Xinjiang Compass Agritechnology Co., Ltd (Changji, China). Genomic DNA was extracted using the standard phenol-chloroform method [28]. During library construction, the genomic DNA was randomly fragmented into approximately 350 bp fragments using a fragmentation machine (Bioruptor® Pico sonication device, Diagenode, Belgium). Subsequently, sequencing adapters were ligated for sequencing following end repair.

### Read mapping and SNP calling

Quality control and filtering were performed using fastp with default parameters [29]. Subsequently, the quality of the reads was assessed using FastQC with default parameters (http://www.bioinformatics.babraham.ac.uk/projects/fastqc). Clean reads were aligned to the *Bos taurus* reference genome ARS-UCD1.2 using the Burrows-Wheeler Aligner BWA-MEM [30]. SAMtools [31] was used to sort the reads, and “MarkDuplicates” in Picard tools (http://broadinstitute.github.io/picard) was used to identify duplicate reads. Genome Analysis Toolkit v4.0 (GATK) [32] were used to detect SNPs. The SNPs were identified using “HaplotypeCaller”, “GenotypeGVCFs” and “SelectVariants” implemented in GATK. Then, “VariantFiltration” was used for hard filtering with the parameters “QD < 2.0 || MQ < 40.0 || FS > 60.0 || SOR > 3.0 || MQRankSum < -12.5 || ReadPosRankSum < -8.0”. PLINK v1.9 [33] was used for filtering with the parameters “--geno 0.2 --maf 0.05 --biallelic-only”. Genotype imputation was conducted using the Beagle software package [34].

### Population structure and genetic analysis

SNPs were pruned using PLINK [33] with the parameter settings “--indep-pair-wise 50 5 0.2”. A principal component analysis (PCA) and ADMIXTURE analysis were conducted using GCTA [35] and ADMIXTURE v1.3 [36] with kinship (K) values from 2 to 13. PLINK provides a pairwise genetic distance matrix (identity-by-state, IBS) for the NJ algorithm. An unrooted NJ tree was constructed with MEGA v11.0 [37] and visualized using EvoView (https://evolgenius.info//evolview-v2/). PCA plots were generated using the ggplot2 package in R v4.2.3.

VCFtools [38] was used to estimate nucleotide diversity in each group with the parameter “--thin 1000”. Linkage disequilibrium (LD) decay with physical distance between SNPs was calculated and visualized by using PopLDdecay [39]. ROHs were identified for estimating homozygosity using PLINK [33] (--homozyg-density 50 --homozyg-gap 100 --homozyg-snp 50 --homozyg-window-het 3 --homozyg-window-missing 5 --homozyg-kb 500 --homozyg-window-snp 50 --homozyg-window-threshold 0.05). The number and length of ROH were estimated and the length of ROH was classified into four categories: 0.5–1 Mb, 1–2 Mb, 2–4 Mb, > 4 Mb. ROH-based inbreeding coefficient (F_ROH_) was measured by PLINK (--het).

### Selective sweep identification

Nucleotide diversity (θπ) in each group was estimated using VCFtools [38] with the parameters “--window-pi 50000 --window-pi-step 20000” and Tajima’s D was estimated with the parameter “--TajimaD 100000” for each group. To explore the selected regions in the genome of Qinchuan cattle, we compared QNC with QCC and set corresponding threshold values (θπ = top 1% (-log10 = 3.26)); Tajima’s D = 0.05; *F*_ST_ = 0.1). We performed comparisons between all cattle breeds using the fixation index (*F*_ST_) with parameter settings “--fst-window-size 50000 --fst-window-step 20000 --weir-fst-pop” using VCFtools. *F*_ST_ values between groups were plotted uusing the pheatmap package in R v4.2.3 [40].

### Gene annotation

A gene annotation file was constructed based on the Ensembl database for annotating candidate genes based on the ARS-UCD 1.2 reference genome. Subsequently, we used a Perl script for annotation based on gene information downloaded from the Ensembl database to identify genes within the selected interval. To gain a better understanding of the gene functions and signaling pathways of the candidate genes, we performed online Gene Ontology (GO) and Kyoto Encyclopedia of Genes and Genomes (KEGG) pathway enrichment analyses using g: Profiler (https://biit.cs.ut.ee/gprofiler/gost) and KOBAS [13] The threshold for significant enrichment was *P* < 0.05. The results of the GO analysis were visualized using an online platform (https://www.bioinformatics.com.cn). A Venn diagram for the genes with overlap among θπ, *F*_ST_ and Tajima’s D analyses was generated using the ggVennDiagram package [41].

### Electronic supplementary material

Below is the link to the electronic supplementary material.

Supplementary Material 1

Supplementary Material 2

Supplementary Material 3

Supplementary Material 4

Supplementary Material 5

Supplementary Material 6

Supplementary Material 7

Supplementary Material 8

Supplementary Material 9

Supplementary Material 10

Supplementary Material 11

## Data Availability

Raw sequencing data are available from the Genome Sequence Archive of the National Genomics Data Center, China National Center for Bioinformation/Beijing Institute of Genomics, Chinese Academy of Sciences (https://bigd.big.ac.cn/gsa/browse/CRA011831).

## Abbreviations

QNC: Qinchuan cattle
QCC: Ancestral Qinchuan cattle
ZSC: Zaosheng cattle
θπ: Nucleotide diversity
F_ST_: Fixation index
NJ: Neighbor-joining
PCA: Principal component analysis
CV: Cross Validation
ROH: Runs of homozygosity
LD: Linkage disequilibrium
GO: Gene Ontology
KEGG: Kyoto Encyclopedia of Genes and Genomes

## References

[1] Berry DP, Conroy S, Pabiou T, Cromie AR. Animal breeding strategies can improve meat quality attributes within entire populations. Meat Sci 2017, 132. 10.1016/j.meatsci.2017.04.019 28515004

[2] Mei C, Wang H, Liao Q, Khan R, Raza SHA, Zhao C, Wang H, Cheng G, Tian W, Li Y (2019). Genome-wide analysis reveals the effects of artificial selection on production and meat quality traits in Qinchuan cattle. Genomics.

[3] Xia X, Zhang S, Zhang H, Zhang Z, Chen N, Li Z, Sun H, Liu X, Lyu S, Wang X (2021). Assessing genomic diversity and signatures of selection in Jiaxian Red cattle using whole-genome sequencing data. BMC Genomics.

[4] Zhang S, Yao Z, Li X, Zhang Z, Liu X, Yang P, Chen N, Xia X, Lyu S, Shi Q (2022). Assessing genomic diversity and signatures of selection in Pinan cattle using whole-genome sequencing data. BMC Genomics.

[5] Taye M, Kim J, Yoon SH, Lee W, Hanotte O, Dessie T, Kemp S, Mwai OA, Caetano-Anolles K, Cho S (2017). Whole genome scan reveals the genetic signature of African ankole cattle breed and potential for higher quality beef. BMC Genet.

[6] Zhang X, Xu H, Zhang C, Bai J, Song J, Hao B, Zhang L, Xia G. Effects of vitamin A on Yanbian Yellow Cattle and their preadipocytes by activating AKT/mTOR signaling pathway and intestinal Microflora. Anim (Basel) 2022, 12(12). 10.3390/ani12121477 PMC9219514 35739812

[7] Wu W, Yu Q-Q, Fu Y, Tian X-J, Jia F, Li X-M, Dai R-T (2016). Towards muscle-specific meat color stability of Chinese Luxi yellow cattle: a proteomic insight into post-mortem storage. J Proteom.

[8] Pei C-X, Mao S-Y, Cheng Y-F, Zhu W-Y (2010). Diversity, abundance and novel 16S rRNA gene sequences of methanogens in rumen liquid, solid and epithelium fractions of Jinnan cattle. Animal.

[9] Wei X, Zhu Y, Zhao X, Zhao Y, Jing Y, Liu G, Wang S, Li H, Ma Y (2022). Transcriptome profiling of mRNAs in muscle tissue of Pinan cattle and Nanyang cattle. Gene.

[10] Yu H, Raza SHA, Pan Y, Cheng G, Mei C, Zan L. Integrative Analysis of Blood Transcriptomics and Metabolomics reveals Molecular Regulation of Backfat Thickness in Qinchuan Cattle. Anim (Basel) 2023, 13(6). 10.3390/ani13061060 PMC10044415 36978600

[11] Hengwei Y, Raza SHA, Wang S, Khan R, Ayari-Akkari A, El Moneim Ahmed DA, Ahmad I, Shaoib M, Abd El-Aziz AH, Rahman SU et al. The growth curve determination and economic trait correlation for Qinchuan bull population. Anim Biotechnol 2022:1–8. 10.1080/10495398.2022.2111309 35980325

[12] Zan L, Wang H, Mei C (2015). Breeding and improvement of Qinchuan cattle and its beef industrialization. J Agricultural Biotechnol.

[13] Bu D, Luo H, Huo P, Wang Z, Zhang S, He Z, Wu Y, Zhao L, Liu J, Guo J (2021). KOBAS-i: intelligent prioritization and exploratory visualization of biological functions for gene enrichment analysis. Nucleic Acids Res.

[14] Kanehisa M, Furumichi M, Sato Y, Kawashima M, Ishiguro-Watanabe M (2023). KEGG for taxonomy-based analysis of pathways and genomes. Nucleic Acids Res.

[15] Estrella NL, Desjardins CA, Nocco SE, Clark AL, Maksimenko Y, Naya FJ (2015). MEF2 transcription factors regulate distinct gene programs in mammalian skeletal muscle differentiation. J Biol Chem.

[16] Wang Y-N, Yang W-C, Li P-W, Wang H-B, Zhang Y-Y, Zan L-S (2018). Myocyte enhancer factor 2A promotes proliferation and its inhibition attenuates myogenic differentiation via myozenin 2 in bovine skeletal muscle myoblast. PLoS ONE.

[17] Wang Y, Mei C, Su X, Wang H, Yang W, Zan L. MEF2A regulates the MEG3-DIO3 miRNA mega cluster-targeted PP2A signaling in bovine skeletal myoblast differentiation. Int J Mol Sci 2019, 20(11). 10.3390/ijms20112748 PMC6600538 31167510

[18] Qin J, Sun Y, Liu S, Zhao R, Zhang Q, Pang W (2019). MicroRNA-323-3p promotes myogenesis by targeting Smad2. J Cell Biochem.

[19] Chen Z-H, Xu Y-X, Xie X-L, Wang D-F, Aguilar-Gómez D, Liu G-J, Li X, Esmailizadeh A, Rezaei V, Kantanen J (2021). Whole-genome sequence analysis unveils different origins of European and Asiatic Mouflon and domestication-related genes in sheep. Commun Biol.

[20] Zhang H, Yu J-Q, Yang L-L, Kramer LM, Zhang X-Y, Na W, Reecy JM, Li H (2017). Identification of genome-wide SNP-SNP interactions associated with important traits in chicken. BMC Genomics.

[21] Han R, Han L, Zhao X, Wang Q, Xia Y, Li H. Haplotype-resolved genome of Sika deer reveals allele-specific gene expression and chromosome evolution. Genomics Proteom Bioinf 2022. 10.1016/j.gpb.2022.11.001 PMC10787017 36395998

[22] Xu Y, Jiang Y, Shi T, Cai H, Lan X, Zhao X, Plath M, Chen H (2017). Whole-genome sequencing reveals mutational landscape underlying phenotypic differences between two widespread Chinese cattle breeds. PLoS ONE.

[23] Stephan W. Selective sweeps. Genetics 2019, 211(1). 10.1534/genetics.118.301319 PMC6325696 30626638

[24] Pavlidis P, Alachiotis N (2017). A survey of methods and tools to detect recent and strong positive selection. J Biol Res (Thessalon).

[25] Nielsen R, Williamson S, Kim Y, Hubisz MJ, Clark AG, Bustamante C (2005). Genomic scans for selective sweeps using SNP data. Genome Res.

[26] Qiu X, Wang H-Y, Yang Z-Y, Sun L-M, Liu S-N, Fan C-Q, Zhu F (2023). Uncovering the prominent role of satellite cells in paravertebral muscle development and aging by single-nucleus RNA sequencing. Genes Dis.

[27] Batut J, Howell M, Hill CS (2007). Kinesin-mediated transport of Smad2 is required for signaling in response to TGF-beta ligands. Dev Cell.

[28] Reid GA: Molecular cloning: A laboratory manual, 2nd edn: by, Sambrook J, Fritsch EF, Maniatis T, Cold Spring Harbor Laboratory Press., 1989. $115.00 (3 vols; 1659 pages) ISBN 0 87969 309 6. In.: Elsevier Current Trends; 1991.

[29] Chen S, Zhou Y, Chen Y, Gu J (2018). Fastp: an ultra-fast all-in-one FASTQ preprocessor. Bioinformatics.

[30] Li H, Durbin R (2009). Fast and accurate short read alignment with Burrows-Wheeler transform. Bioinformatics.

[31] Li H, Handsaker B, Wysoker A, Fennell T, Ruan J, Homer N, Marth G, Abecasis G, Durbin R (2009). The sequence Alignment/Map format and SAMtools. Bioinformatics.

[32] Nekrutenko A, Taylor J (2012). Next-generation sequencing data interpretation: enhancing reproducibility and accessibility. Nat Rev Genet.

[33] Purcell S, Neale B, Todd-Brown K, Thomas L, Ferreira MAR, Bender D, Maller J, Sklar P, de Bakker PIW, Daly MJ (2007). PLINK: a tool set for whole-genome association and population-based linkage analyses. Am J Hum Genet.

[34] Browning SR, Browning BL (2007). Rapid and accurate haplotype phasing and missing-data inference for whole-genome association studies by use of localized haplotype clustering. Am J Hum Genet.

[35] Yang J, Lee SH, Goddard ME, Visscher PM (2011). GCTA: a tool for genome-wide complex trait analysis. Am J Hum Genet.

[36] Alexander DH, Lange K (2011). Enhancements to the ADMIXTURE algorithm for individual ancestry estimation. BMC Bioinformatics.

[37] Tamura K, Stecher G, Kumar S (2021). MEGA11: Molecular Evolutionary Genetics Analysis Version 11. Mol Biol Evol.

[38] Danecek P, Auton A, Abecasis G, Albers CA, Banks E, DePristo MA, Handsaker RE, Lunter G, Marth GT, Sherry ST (2011). The variant call format and VCFtools. Bioinformatics.

[39] Zhang C, Dong S-S, Xu J-Y, He W-M, Yang T-L (2019). PopLDdecay: a fast and effective tool for linkage disequilibrium decay analysis based on variant call format files. Bioinformatics.

[40] Diao C, Xi Y, Xiao T (2018). Identification and analysis of key genes in osteosarcoma using bioinformatics. Oncol Lett.

[41] Gao C-H, Yu G, Cai P (2021). ggVennDiagram: an intuitive, easy-to-Use, and highly customizable R Package to Generate Venn Diagram. Front Genet.

